# Potential of feeding beef cattle with whole corn crop silage and rice straw in Malaysia

**DOI:** 10.1007/s11250-018-1538-2

**Published:** 2018-02-17

**Authors:** Muhamad Hazim Nazli, Ridzwan Abdul Halim, Amin Mahir Abdullah, Ghazali Hussin, Anjas Asmara Samsudin

**Affiliations:** 1 Department of Crop Science, Faculty of Agriculture, Universiti Putra Malaysia, 43400 Serdang, Selangor Malaysia; 2 Department of Agribusiness, Faculty of Agriculture, Universiti Putra Malaysia, 43400 Serdang, Selangor Malaysia; 3 Livestock Science Research Centre, Malaysian Agriculture Research and Development Institute, 43400 Serdang, Selangor Malaysia; 4 Department of Animal Science, Faculty of Agriculture, Universiti Putra Malaysia, 43400 Serdang, Selangor Darul Ehsan Malaysia

**Keywords:** Corn silage, Rice straw, Beef cattle, Growth performance, Intake, Digestibility

## Abstract

The potential of using whole corn crop silage and rice straw as an alternative feed for the beef cattle based on the intake and growth performance were evaluated. Using randomised completely block design, nine adult Mafriwal cattle were blocked intro three groups and treated with three different forage diets supplemented with 20% pelleted palm kernel cake on dry matter basis. The treatments were 100% rice straw (RS), 100% corn silage (CS) and an equal mixture of rice straw and corn silage (MIX) fed ad libitum. The animals were housed in individual pens, and the feeding trial was conducted for 12 weeks with 2 weeks of adaptation period. The results showed that CS had the best feed nutritive composition with the lowest concentration of highly indigestible fibre and the highest concentration of organic matter and energy. The CS also had the highest intake, and the corn silage inclusion in MIX managed to improve the intake on par with CS in terms of the dry matter intake of body weight (DMI of BW), voluntary intake (VI) and crude protein (CP) intake. Cattle fed with CS gave the highest and most stable BW gain with an average daily gain (ADG) of 808 g/day rivalling cross-bred cattle fed with high amount of concentrates. The all straw diet (RS) supplemented with PKC recorded a positive ADG of 133 g/day while the MIX gave 383 g/day matching total Napier grass diet.

## Introduction

Beef production in Malaysia met only 22.4% of the national requirement with per capita consumption of 7.06 kg/year in 2016 (DVS 2017). In recent years, consumption has increased more than production thus causing a decrease in self-sufficiency and more imported beef entering the market (Said and Man 2014). As the industry has always been dominated by small and medium scale farmers with limited grazing land and resources, the industry is dependent on expensive purchased concentrates (Ariff et al. 2015). Feed represents almost 70% of the production cost, and cheaper alternative feeds are needed to help the farmer overcome the increasing feed prices and cheaper imported meat. This problem can be resolved by producing the feed locally as the quality and quantity can also be manipulated accordingly.

Forage resources play a very important role in ruminant feeding in tropical countries, and in Malaysia, livestock nutrition depends on natural or cultivated forages (Najim et al. 2015). Depending on the wet or dry season, tropical grasses are inconsistent in terms of quantity and quality. The diminishing pasture area forced farmers to rely more on concentrates than roughage thus increasing the production cost (Rahman et al. 2014).

Currently, corn silage is the major source of conserved forage in Europe and North America (Alonso et al. 2013). However, production in Malaysia is very low until today, and no formal production data are available. Recently, there is an increasing trend for silage production which is mostly made from local grasses and corn stover. Whole corn crop silage can be an alternative feed resource as it is considered the best among non-legume forage crops (Chaudhary et al. 2014) and can be heavily mechanised thus reducing the production cost. A local study by Khaing et al. (2015) in goats showed that substitution of the Napier grass with corn silage improved the weight gain, feed intake and digestibility. In addition, the concentrate requirement can be reduced by corn silage inclusion into the ration (Keady et al. 2013). Apart from corn silage, by-product such as rice straw can also be used as an alternative feed as it is abundant and cheap, though rice straw alone does not provide enough nutrients for high production ruminants (Liu et al. 2015).

In view of these facts, swift action must be taken to avoid the repeat of heavy imported feed dependence in broiler and swine industry that had caused corn to be nation’s biggest import item in term of quantity in recent years (FAOSTAT 2017). In addition, very limited data are available as only few researches have been carried out on cattle performance when fed with either corn silage or rice straw in Malaysia. Clearly, there are information gaps and with the nation’s ‘feed security’ at risk, the present study was done as a baseline study for further improvement in Malaysia. The objective of the study was to determine the potential of feeding whole corn crop silage and rice straw on the intake and growth performance of beef cattle.

## Materials and methods

### Animal welfare and ethics

This study was carried out following the guidelines of the research policy of the Universiti Putra Malaysia (UPM) on animal welfare and ethics. The care of the experimental cattle was in accordance to Malaysian Standards.

### Experimental animals and feeding

The experiment was conducted at the Institute of Tropical Agriculture (ITA) Animal Facility, UPM Serdang, Selangor. Using a randomised complete block design (RCBD), nine Mafriwal (Malaysian Friesian-Sahiwal) cattle were used and blocked into three groups with mean body weights of 116 kg (Block 1), 136 kg (Block 2) and 148 kg (Block 3). The cattle were fed with different level of rice straw and corn silage mixture that comprised 80% of the total feed (Table 1). The treatments were assigned randomly within each block. The remaining 20% portion consists of PKC pellet to meet the animal minimum feed requirement. The feed requirement was calculated based on 3% of the animal live weight with daily adjustment based on the individual animal intake and given ad libitum.

**Table 1:** Treatment group forage ratio

Treatment	Forage ratio (80%)
RS	100% rice straw
MIX	50:50 rice straw and corn silage
CS	100% corn silage

The corn plant was planted in Field 15, UPM and harvested at the dent stage. The plant was chopped and packed in plastic drums with a minimum storage period of 30 days. The rice straw was bought from paddy plantation area in Kuala Selangor and covered with polyethylene sheets to prevent damage. The animals were dewormed and given 14 days-adaptation period prior to the commencement of the experiment. The feeding trial then commenced for 84 days with the digestibility trial conducted concurrently in the final week. The individual feed was prepared daily and fed in the morning and evening with the rice straw chopped for easy handling and better mixing. The feed refused was removed daily with mineral blocks, and continuous fresh water were made available without restrictions. The amount of feed offered and refused was recorded daily to calculate the daily feed intake while weighing was done every 2 weeks before feeding time to obtain the weight changes.

### Analytical technique

During 7 days of digestibility trial, representative feed and faecal samples were collected, weighed and oven-dried at 60 °C for 48 h. The samples were then ground to pass through 1-mm screen and stored in pill boxes for further analysis. The nutritive values were determined by FOSS DS2500 Near Infrared Spectroscopy (NIRS) with additional calibration from the feed and faecal samples analysed using standard laboratory procedure. The CP was measured based on the method by AOAC (2000) while the neutral detergent fibre (NDF) and acid detergent fibre (ADF) were determined using FOSS FiberCap 2023 System (ISO 13906 2008). The lignin determination was done using FOSS Fibertec M6 1020/1021 system (ISO 13906 2008). The energy values were calculated using the formulae below based on the publication by NRC (2000).

$$ \mathrm{Digestible}\ \mathrm{energy},\mathrm{DE}\ \left(\mathrm{MJ}/\mathrm{kg}\right)=\left[0.04409\ \mathrm{x}\ \mathrm{TDN}\ \left(\%\right)\right]\ \mathrm{x}\ 4.184 $$

$$ \mathrm{Metabolizable}\ \mathrm{Energy},\mathrm{ME}\ \left(\mathrm{MJ}/\mathrm{kg}\right)=\left[\mathrm{DE}\ \mathrm{x}\ 0.82\right]\ \mathrm{x}\ 4.184 $$

Nutrient digestibility was calculated by dividing the differences between the feed and faecal composition values with the feed nutritive value, then converted into percentage values. Based on the animal’s intake and BW gain, the feed conversion efficiency (FCE) and feed conversion ratio (FCR) were calculated. The average daily gain (ADG) was calculated by dividing the initial and final live weight differences with the total number of experimental days. Based on the daily feed offered and refused records, the daily DM feed intake was calculated by dividing the total feed eaten by the animals (based on the DM content) with the total number of experimental days. The voluntary intake (VI) was calculated using the formula below:

$$ \mathrm{VI}\ \left(\mathrm{g}/{\mathrm{kg}}^{0.75}\right)=\mathrm{Average}\ \mathrm{DM}\ \mathrm{intake}\ \left(\mathrm{kg}\right)\ \mathrm{x}\ 1000/\mathrm{Average}\ \mathrm{BW}\ {\left(\mathrm{kg}\right)}^{0.75} $$

### Statistical analysis

The data were presented in mean with the standard error and analysed using analysis of variance (ANOVA). Any significant differences between the treatment means were compared using Least Significance Difference (LSD) test of *P* value ≤ 0.05. The analysis was done using SAS version 9.4. The mathematical model assumption used was:

$$ {Y}_i=\mu +{T}_i+{\beta}_i+{\varepsilon}_i $$

where *Y*_*i*_ is the dependent variable (ADG; intake; nutrient content; feed digestibility etc.), *μ* is the overall mean, *T*_*i*_ is the *i*th treatment (RS; MIX; CS) effect, *β*_*i*_ is the *i*th block effect and *Ɛ*_*i*_ is the residual error of the *i*th observation.

## Results

All the feed composition parameters showed significant difference among treatments except for the NDF content (Table 2). RS had the highest DM, ADF, cellulose and ash contents and the lowest CP, OM, hemicellulose and lignin contents while CS had the highest OM and hemicellulose contents and the lowest DM, ADF, cellulose, ash and lignin contents. All the energy value parameters were significantly different with RS having the lowest values while MIX and CS were not significantly different (Table 2).

**Table 2:** Feed treatment nutritive and energy composition on dry matter basis

	Treatment			SEM	*P* value
	RS	MIX	CS		
Nutritive value (%)					
DM	89.3^a^	74.9^b^	56.3^c^	4.97	0.006**
CP	11.2^c^	14.3^a^	12.6^b^	0.45	0.002**
OM	83.1^c^	85.4^b^	88.5^a^	0.80	< 0.001**
NDF	80.8^a^	80.9^a^	81.4^a^	0.28	0.666^ns^
ADF	58.9^a^	40.2^b^	42.5^b^	3.06	0.005**
Lignin	1.8^b^	3.5^a^	2.5^b^	0.29	0.016*
Hemicellulose	21.9^b^	40.7^a^	39.0^a^	3.07	0.001**
Cellulose	40.2^a^	22.1^b^	28.5^b^	2.74	0.005**
Ash	17.0^a^	14.6^b^	11.5^c^	0.80	< 0.001**
Energy value (MJ/kg)					
DE	7.78^b^	10.4^a^	10.6^a^	0.46	0.002**
ME	6.38^b^	8.48^a^	8.72^a^	0.38	0.002**
NE_M_	2.87^b^	4.93^a^	5.15^a^	0.37	0.002**
NE_G_	0.65^b^	2.58^a^	2.78^a^	0.35	0.002**

Means with different superscript letters in each parameter are significantly different

*RS* 100% rice straw, *MIX* 50% rice straw + 50% corn silage, *CS* 100% corn silage, *SEM* standard error of mean, *DM* dry matter, *CP* crude protein, *OM* organic matter, *NDF* neutral detergent fibre, *ADF* acid detergent fibre, *DE* digestible energy, *ME* metabolizable energy, *NE*_*M*_ net energy for maintenance, *NE*_*G*_ net energy for gain

^ns^Not significant at *P* > 0.05, *significance level at *P* < 0.05, **significance level at *P* ≤ 0.01

Significant differences (*P* ≤ 0.01) were also observed among the treatments in all the intake parameters (Table 3). CS had the highest value for total feed intake, daily feed intake, total DMI, daily DMI, OM and NDF intakes. But for DMI of BW, VI and CP intakes, both CS and MIX showed no significant difference. While for RS, all the intake values were the lowest among the treatments.

**Table 3:** Feed intakes of the different feed treatments

	Treatment			SEM	*P* value
	RS	MIX	CS		
Total FI (kg)	289^c^	573^b^	917^a^	96	0.001**
Daily FI (kg/day)	3.4^c^	6.8^b^	10.9^a^	1.14	0.001**
Total DMI (kg)	258^c^	431^b^	511^a^	40	0.001**
Daily DMI (kg/day)	3.1^c^	5.1^b^	6.1^a^	0.48	0.001**
DMI of BW (%)	2.2^b^	3.2^a^	3.0^a^	0.17	0.044*
VI (g/kg^0.75^)	97.6^b^	133^a^	138^a^	7.3	0.003**
OM intake (kg/day)	2.6^c^	4.4^b^	5.4^a^	0.45	0.001**
CP intake (kg/day)	0.34^b^	0.74^a^	0.76^a^	0.07	0.004**
NDF intake (kg/day)	2.5^c^	4.2^b^	5.0^a^	0.40	0.001**

Means with different superscript letters in each parameter are significantly different

*RS* 100% rice straw, *MIX* 50% rice straw + 50% corn silage, *CS* 100% corn silage, *SEM* standard error of mean, *FI* feed intake, *DMI* dry matter intake, *BW* body weight, *VI* voluntary intake, *OM* organic matter, *CP* crude protein, *NDF* neutral detergent fibre

^ns^Not significant at *P* > 0.05, *significance level at *P* ≤ 0.05, **significance level at *P* ≤ 0.01

**Fig. 1:**
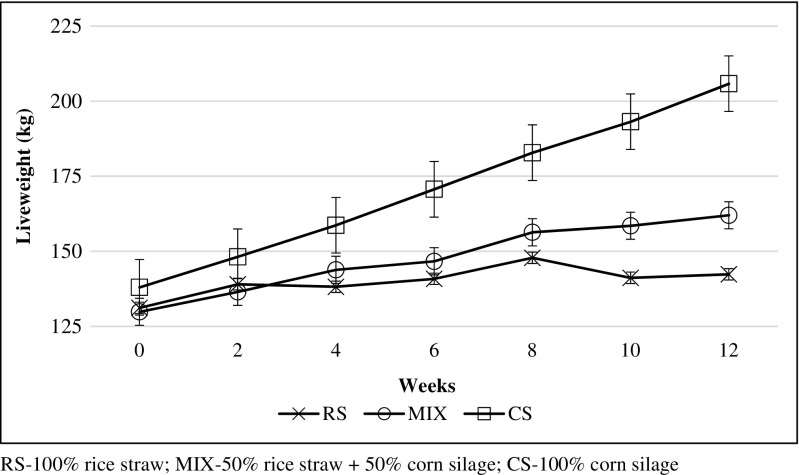
Increase in weight of cattle fed rice straw and corn silage

The initial BW was similar across the treatments while for the final BW, total BW gain and ADG, CS had the significantly highest values of 206, 67.8 and 808 g/day respectively (Table 4). In contrast, RS had the lowest value of all the parameters except for the final BW that was not significantly different from MIX. Furthermore, CS had the highest increment with the most linear response among the treatments while RS fluctuated heavily over the trial period even though it has higher increment than MIX early in the trial (Fig. 1).

**Table 4:** Weight changes, feed digestibility and feed efficiency values of the different feed treatments

	Treatment			SEM	*P* value
	RS	MIX	CS		
Animal weight change					
Initial BW (kg)	131^a^	130^a^	138^a^	5.6	0.684^ns^
Final BW (kg)	142^b^	162^b^	206^a^	12	0.010**
Total BW gain (kg)	11.2^c^	32.2^b^	67.8^a^	8.62	0.001**
ADG (g/day)	133^c^	383^b^	808^a^	103	< 0.001**
Feed digestibility (%)					
DM digestibility (%)	74.7^a^	77.5^a^	74.7^a^	0.93	0.488^ns^
CP digestibility (%)	56.9^b^	64.7^a^	56.8^b^	1.35	0.003**
DDM (%)	43.0^b^	57.6^a^	55.8^a^	2.39	0.005**
TDN (%)	42.2^b^	56.1^a^	57.7^a^	2.52	0.002**
Feed efficiency value					
FCE	0.04^c^	0.08^b^	0.13^a^	0.01	< 0.001**
FCR	26.0^a^	13.3^ab^	7.6^b^	3.27	0.049*
RFV	102^b^	230^a^	262^a^	26	0.003**
RFQ	46.9^b^	56.4^a^	60.0^a^	2.06	0.002**

Means with different superscript letters in each parameter are significantly different

*RS* 100% rice straw, *MIX* 50% rice straw + 50% corn silage, *CS* 100% corn silage, *SEM* standard error of mean, *BW* body weight, *ADG* average daily gain, *DDM* digestible dry matter, *TDN* total digestible nutrient, *FCE* feed conversion efficiency, *FCR* feed conversion ratio, *RFV* relative feed value, *RFQ* relative feed quality

^ns^Not significant at *P* > 0.05, *significance level at *P* ≤ 0.05, **significance level at *P* ≤ 0.01

There were significant differences (*P* < 0.01) among the treatments for the CP digestibility, DM digestibility (DDM) and total digestible nutrient (TDN) while no significant difference was observed in the DM digestibility (Table 4). Both MIX and CS had the highest DDM and TDN values while RS had the lowest value, but there was no significant difference in the CP digestibility between RS and CS. Significant differences were observed in all the efficiency parameters. RS had the lowest FCE, RFV and RFQ values while both MIX and CS had the highest RFV and RFQ values.

## Discussion

### Feed composition

Corn silage superiority was demonstrated by the high CP and OM content with a lower concentration of ADF, cellulose, lignin and ash (Table 2). Compared to PKC diet, CS had less ADF with almost similar NDF value (Kum and Wan Zahari 2011). RS had higher than normal CP with lower lignin content, but this variation was expected as various agronomic practices and weather condition play major roles in influencing rice straw nutritive value (Rosmiza et al. 2015). With 50% inclusion of corn silage in MIX, the CP and OM amounts increased significantly while the ADF, cellulose and ash contents decreased thus improving the lower quality rice straw portion. In addition, the energy values also improved significantly and were not significantly different from CS (Table 2). The lower DE value of RS was most likely due to rice straw having a higher amount of the least digestible fibre (ADF and cellulose). The CS ME value was less than that reported by Khaing et al. (2015) due to higher fibre portion caused by the differences in the variety and harvest stage. However, the value was higher compared to Napier grass (Rahman et al. 2014) that is commonly used by Malaysian farmers. As expected, the net energy (NE) for gain is lower than NE for maintenance because the feed energy was used more efficiently for maintenance than gain by the animals. Compared to DE, RS was only able to give a mere 8% of the energy to the animal gain while MIX and CS gave 25 and 26% accordingly. This shows more energy is available in corn silage usage and was evident from the animals more active behaviour.

### Feed intake

Clearly CS was the most palatable as most of the intake values were far superior than RS and MIX (Table 3). The inclusion of corn silage in MIX promotes higher intake and consequently better animal performance (Zaralis et al. 2014). Corn silage has higher intake values because of the higher starch content with less indigestible fibre portion. The significant increase in the NDF intake when fed with corn silage was due to the silage having an abnormally high NDF. Even though the NDF was similar to the rice straw, surprisingly, the other intake parameters were not negatively affected. RS had the lowest intake value because rice straw had the most indigestible fibre concentration among the treatments. The inclusion of corn silage improved the feed palatability in terms of the dry matter intake (DMI) of body weight, voluntary intake and CP intake. Corn silage improved the rice straw mixture intakes at 50% inclusion level. The MIX DMI of BW, VI and CP intake increased significantly and on par with 100% corn silage diet in CS. The corn silage intake was higher than cattle fed with fresh Napier grass (Siddque et al. 2015) and Napier grass silage (Bureenok et al. 2012). The higher CP intake is important for beef cattle production as it greatly influences animal growth performance. As the DMI of BW increased, the animal intake was parallel with the theoretical feed calculation (3% of the BW) causing less feed refused or wastage. The intake improvements show corn silage flexibility when mixed with lower quality feed, and this is useful when corn silage is limited or too expensive.

### Live weight changes

The initial BW showed no significant difference among the treatments indicating that blocking and randomisation were done correctly to prevent bias among the treatments (Table 4). No significant difference was observed between RS and MIX final BW but there was a significant difference in the ADG. This was due to ADG calculation that includes the initial BW, thus giving a more precise indicator for animal performance. As expected, CS had the highest ADG value, close to cattle fed with 80% PKE concentrates (Kum and Wan Zahari 2011). The 808 g/day ADG was also competitive compared with other cattle breed using conventional feed in Malaysia’s feedlot system; Charolais-KK (816 g/day), Simmental-KK (750 g/day) and Limousin-KK (750 g/day) (Johari and Jasmi 2009). The CS live weight increase was also stable compared to RS and MIX that experienced heavier fluctuations, possibly due to stress from the environmental changes (Fig. 1). The inclusion of rice straw in MIX reduced the ADG significantly, but it was significantly higher than from the all-rice straw diet. The corn silage inclusion can still give a competitive ADG compared to fresh Napier grass (Lapitan et al. 2008). Feeding cattle with crop waste rice straw can also give a positive ADG, though the gain should also be attributed to the addition of PKC.

### Feed digestibility and efficiency

Corn silage inclusion in MIX significantly increased the DDM and TDN values, providing more available digestible nutrient that can increase the growth potential (Table 4). Even though the DM and CP digestibility was similar between RS and CS, CS had more CP and digestible components signalling higher feeding potential. Compared to high quality PKC, all the treatments had similar DM digestibility but lower CP digestibility (Kum and Wan Zahari 2011). The corn silage CP and DM digestibility were also higher than Napier grass diet (Khaing et al. 2015) while the TDN value was higher than conventional Napier grass (Nurjana et al. 2016). As the ADG increased significantly with corn silage inclusion, the FCE and FCR also improved significantly while the increasing DMI was responsible for the better RFV and RFQ values. When fed 100% corn silage, the FCR value was better than the 80% PKC with oil palm frond (Wan Zahari et al. 2003). However, when compared to research by Addah et al. (2011) in the continental climate of western Canada, the FCE values of CS were much lower. The difference was probably due to the various external factors such as the climate and the animal’s genetic makeup. The higher CS DM content might also contribute to the difference as increasing DM can cause a significant ADG decrease (Hilscher et al. 2016).

## Conclusion

In conclusion, the corn silage diet fed to beef cattle had very good nutritive value, with high intake and BW gain. Corn silage has the potential to be fed as the basal diet for high producing beef cattle as the ADG of 808 g surpassed most conventional feeds in Malaysia. A mixture of corn silage and rice straw also showed promising results with nutritive values, intake and BW gain comparable to conventional feeding with Napier grass. Although inclusion of rice straw in the diet reduced intake and BW gain, it is particularly useful when high quality feeds are limited or too expensive. But rice straw cannot be used as a complete ration, only as a replacement for roughage or in this case, fed with concentrates.
